# Effects of X-Ray Dose On Rhizosphere Studies Using X-Ray Computed Tomography

**DOI:** 10.1371/journal.pone.0067250

**Published:** 2013-06-26

**Authors:** Susan Zappala, Jonathan R. Helliwell, Saoirse R. Tracy, Stefan Mairhofer, Craig J. Sturrock, Tony Pridmore, Malcolm Bennett, Sacha J. Mooney

**Affiliations:** 1 Division of Agricultural and Environmental Sciences, University of Nottingham, Sutton Bonington, United Kingdom; 2 School of Computer Science, University of Nottingham, Nottingham, United Kingdom; 3 Division of Plant and Crop Sciences, University of Nottingham, Sutton Bonington, United Kingdom; University of Zurich, Switzerland

## Abstract

X-ray Computed Tomography (CT) is a non-destructive imaging technique originally designed for diagnostic medicine, which was adopted for rhizosphere and soil science applications in the early 1980s. X-ray CT enables researchers to simultaneously visualise and quantify the heterogeneous soil matrix of mineral grains, organic matter, air-filled pores and water-filled pores. Additionally, X-ray CT allows visualisation of plant roots in situ without the need for traditional invasive methods such as root washing. However, one routinely unreported aspect of X-ray CT is the potential effect of X-ray dose on the soil-borne microorganisms and plants in rhizosphere investigations. Here we aimed to i) highlight the need for more consistent reporting of X-ray CT parameters for dose to sample, ii) to provide an overview of previously reported impacts of X-rays on soil microorganisms and plant roots and iii) present new data investigating the response of plant roots and microbial communities to X-ray exposure. Fewer than 5% of the 126 publications included in the literature review contained sufficient information to calculate dose and only 2.4% of the publications explicitly state an estimate of dose received by each sample. We conducted a study involving rice roots growing in soil, observing no significant difference between the numbers of root tips, root volume and total root length in scanned versus unscanned samples. In parallel, a soil microbe experiment scanning samples over a total of 24 weeks observed no significant difference between the scanned and unscanned microbial biomass values. We conclude from the literature review and our own experiments that X-ray CT does not impact plant growth or soil microbial populations when employing a low level of dose (<30 Gy). However, the call for higher throughput X-ray CT means that doses that biological samples receive are likely to increase and thus should be closely monitored.

## Introduction

### Dose to Sample in X-ray CT Investigations

X-ray Computed Tomography (CT) is a non-destructive imaging technique commonly used to observe and quantify aspects of the soil environment including plant root development [1], [2], [3], [4], fungal influences [5], [6], changes to pore structure [7] and the influence of microbial activity [8]. One often overlooked aspect of X-ray CT studies involving soil is the influence of X-ray dose on the biological subject of interest (e.g. plants and animals) in these studies. For those studies that have included unscanned controls and reported X-ray dose or parameters enabling calculation of dose, there has been no discernible influence on plant root growth [1], [9], fungal [5] or microbial activity [10]. However, it should be noted that these samples have received relatively small X-ray dose to sample (i.e. <1.5 Gy). With the advent of higher throughput X-ray CT techniques [11], which often involve multiple scans of the same sample over longer periods of time, total dose and therefore potential influence of the received dose will increase for individual samples. Stuppy et al. [12] argued that X-ray CT was not feasible for living systems due to repeated exposure to X-rays. However, Dutilleul et al. [13] stated that given the right precautions to limit and assess dose effects, X-ray CT is suitable for repeated observation of living organisms and particularly plants.

Dose is the quantity of energy absorbed by an object after exposure to radiation, making it a critical factor for consideration in X-ray CT studies and thus something that should be closely monitored [9], [14]. However, many of the previously published X-ray CT studies do not report X-ray dose or provide insufficient information to calculate dose and its subsequent impact on plant growth or microbial activity. X-ray dose is estimated from tube current, voltage, exposure time and distance (r) from source, and has an exponential relationship to the distance between the X-ray source and the sample, as described by Gauss’ Law. Dose decreases in air by 1/r^2^, making source to sample distance a critical determinant of intensity. This ratio was found to be true for energies between 50kV and 150kV and with currents between 1 µA and 400 µA [15], which is consistent with most recent X-ray CT investigations involving soil and plants. Filters can influence the dose received by the sample, by progressively attenuating the highest and lowest X-rays, producing a narrower spectrum X-ray beam. Some filters composed of metals with low attenuation have little effect on dose. For example, Stupian [15] observed no effect on radiation dose using a 1.6 mm aluminium filter. Furthermore sample composition and size play a key role in resultant doses. Common artefacts such as streaks and ‘shadowing’ behind the constituent of interest occur during photon starvation, when X-rays have insufficient velocities to penetrate certain sample constituents [16], [17]. Likewise container composition and thickness can play a critical role in shielding samples from X-ray exposure [18], and so need to be carefully evaluated before dose calculations can be applied.

The accurate calculation of dose is notoriously difficult due to the complex nature of radiation interaction with matter. For example, X-rays can interact with matter in several ways that are themselves difficult to predict. Primarily, X-ray attenuation by a material is determined by processes such as absorption, scattering, refraction and reflection, as well as magnetic interactions, although these are quite rare [19]. In an effort to quantify the minimum dose required to acquire a tomographic image, Jenneson et al. [20] describe a method for estimating these X-ray interactions, and thus dose to the centre of a sample as:
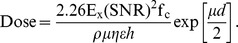
where E_x_ is the beam energy; SNR the signal-to-noise ratio; f_c_ the Compton factor (normalisation parameter reflecting photon energy after inelastic scattering [19]); *ρ* the density of the sample; *µ* the attenuation coefficient; 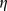 the detector efficiency; 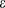 the planar pixel size; *h* the slice thickness; *d* the diameter of the reconstruction. However, it is unusual for many of these measures to be reported in publications. In an effort to establish actual dose rather than an estimation, Stupian [15] measured dose directly with a Radcal® 2026°C ionisation chamber meter. Whilst this is preferable as it gives a near-instantaneous reading of dose at the sample boundary, it relies on laboratories using X-ray CT scanners to have the required equipment. A simpler indication of dose can be made using freely available online calculators such as the Rad Pro Dose Calculator [21]. Using X-ray empirical data from British Standard BS 4094-2∶1971 (Recommendation for data shielding from ionizing radiation – Part 2: Shielding from X-radiation), all that is required for the estimation of sample dose is a basic understanding of the scanning parameters and filters utilised. When we sought to estimate dose from previously published work it was often necessary to infer sample to X-ray source distance from the scanners used because the actual distance was not provided. Furthermore filter thickness and material were commonly unreported, but have a large influence on the resultant X-ray exposure of samples.

### X-ray Dose and Plants

#### Growth of plants exposed to X-rays before germination

X-ray studies involving seeds (imbibed and dry) from 70 plant species showed that exposure to moderate X-ray sources (0.01 Gy to 5 Gy) had a positive influence on shoot and root elongation [14], as well as increased branching in Colorado wild potato (Solanum jamesii) [22]. At higher doses (>15 Gy), significant reduction of seed germination, shoot and root growth, budding, flowering and fruiting were identified in many plant species including field bean (Phaseolus vulgaris) [23] and Nicotiana tabacum [24]. Additionally, the influence of X-ray exposure on plant growth is highly dependent on plant type as well as variety. Sunflowers (Helianthus L.) displayed negative growth effects when the imbibed seeds were exposed to doses greater than 33 Gy [14]. In field bean, doses as low as 26 Gy produced inhibition of germination and chlorophyll abnormalities [23]. However, much lower doses (0.05 Gy) impaired germination of date palm (*Phoenix dactylifera* L.), reduced DNA production, altered biosynthesis of plant pigment (chlorophyll a, chlorophyll b and xanthophylls) and negatively impacted root and shoot growth of the germinated seeds [25].

#### Growth of plants exposed to X-rays after germination

**Figure 1:**
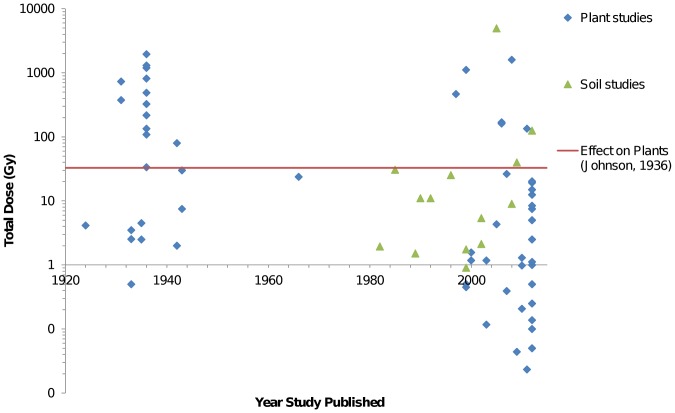
X-ray dose in plant and soil studies. Dose was calculated in Rad Pro with X-ray parameters derived from literature. Most studies involve doses below the 33Gy threshold noted by Johnson [14], below which she did not observe visible alteration of post-germination plant growth after X-ray exposure. Note the reduction in published studies between 1940 and 1980 and the clear rise in the 1990s.

In comparison to plants exposed as seeds, plants are less susceptible if exposed to X-rays post-germination. However, as noted previously the impact of X-ray exposure is highly variable and dependent on the plant type, variety and developmental stage. Dhondt et al. [26] reported growth inhibition in repeatedly scanned Arabidopsis thaliana L. seedlings, which is consistent with the observations of Johnson [14] that X-ray exposure at seedling stage often had a negative influence on growth. However, no indication of X-ray energy was provided by Dhondt et al. [26], so dose could not be calculated from the manuscript. Many of the early experiments investigating plant-dose response involved doses that were several orders of magnitude greater than that considered lethal to humans, an acute dose of >4 Gy [27]. However, these doses do not necessarily impact plant growth and microbial activity, as doses below 33 Gy showed little impact on plant growth [14]. Since 2003, most experiments have involved X-ray exposure equivalent to less than 1.5 Gy (Figure 1), largely as a result of optimised detector response and improvements to acquisition methodologies [28].

### The Influence of X-ray Radiation on Soil Microbial Populations

The irradiation of soil could influence microbial communities through the direct ionisation of cells causing DNA mutation, and the indirect radiolysis of cell water creating damaging free radicals within extra- and intra-cellular fluids [29]. Free radicals can cause single or double stranded DNA breaks [30], damaging future cell and plant development. Yet, to date there has been little work to assess the impact of X-ray radiation on soil constituents, with much of the focus being based on γ-rays due to its application in soil sterilisation procedures. Jackson et al. [29] demonstrated that fungi are more sensitive to radiation than bacteria, with γ-irradiation doses as low as 10 Gy able to alter fungal populations. Responses to radiation continually change as enzymatic activity in soils aid recovery from acute doses, although sensitivity is dependent on a large range of physiological factors such as metabolic activity, organism size and complexity, and life-cycle stage [30]. A dose of 10 Gy of X-ray radiation is equivalent to 10 Gy of γ-ray radiation since X-rays and γ-rays have the same radiation weighting factor, formerly known as quality factor, which is a measure of the expected biological impact of ionising radiation often used in radiation protection [19]. For example, α-particles have a quality factor of 20, meaning on average α-particles are expected to produce 20 times the biological damage of X-rays or γ-rays. Furthermore soil moisture status plays a key role in indirect radiation damage. In wetter soils the ability to form free radicals through the radiolysis of water is increased, requiring a lower dose to harm microbial populations than dry soils [29]. These findings are consistent with those of McNamara et al. [30], who in a meta-analysis of published results suggested that higher irradiation doses may be required to eliminate bacteria in dry soils. Due to the heterogeneity of soil systems, it still remains a challenge to accurately define the impact of radiation of soil-borne populations, although γ-ray doses reported as having impacts on soil constituents are an order of magnitude greater than any found in modern X-ray CT studies.

To date, there is controversy surrounding the expected range of X-ray doses thought to impact plant and soil samples analysed via X-ray CT. We aimed to summarise key sources that report the influence of X-ray dose on plant or soil samples, particularly those involving X-ray CT. In addition, two experiments were completed that aimed to assess the potential effect of dose received during X-ray CT on (i) plant growth and (ii) soil microbial activity.

## Materials and Methods

### Data Collection

Web of Knowledge (Institute of Scientific Information) was utilised as a search database to find related publications with the search term “plant AND X-ray Computed Tomography” or “soil AND X-ray Computed Tomography”. This identified relevant literature dating back 30 years, 93 plant related publications and 346 soil related works. Papers from earlier than 1982 were found through hard copies at the University of Nottingham library (66 items). From the 505 publications identified during the search, 320 were excluded because they did not pertain to plants, soil, X-ray CT or X-ray exposure. From the remaining 185 documents, a database containing data for X-ray energy, current, distance between source and sample, filter utilised, scan timing, total exposure time, sample type and date of publication was created. The 59 publications without information about X-ray energy, current or scan time were excluded from analysis. Reported dose to sample was recorded if provided and compared to Rad Pro X-ray Dose Calculator results, to validate the assumptions made using online tools. In total 126 publications were included in the dose assessment (Figure S1). The range of calculated X-ray doses from each publication was included in Figure 1.

### Dose Calculations Derived from the Literature

Doses were estimated using the Rad Pro Dose Calculator. Due to restrictions of the dose calculator, X-ray doses were calculated assuming a 30 cm source-detector distance for industrial scanners and 100 cm distance for medical scanners. A 1 mm Be filter was used for the calculation if no data was given in the reviewed literature, as this was the minimum available shielding in the Rad Pro software and hence represents a worst case scenario.

Ultimately, all dose calculations are predictions, rather than actual measurements, due to the inherent randomness of X-ray energy and its interaction with materials. X-ray doses in this manuscript are expressed as absorbed dose, which reflects the energy absorbed per unit mass for any radiation source and any material. The concept of absorbed dose was adopted by the International Commission on Radiological Protection (ICRP) to enable comparison of potential radiobiological impact from all types of radiation and a variety of sample types. Absorbed dose best meets the goals of the manuscript to provide a means for dose comparison in plant-soil studies involving X-ray CT. Effective dose cannot be used here as it includes a weighting for the sample composition, which is extremely heterogeneous amongst soil-plant-microbe samples, even in replicates using the same soil type and treatment.

### Soil and Plant Sample Preparation and Treatment

Polypropylene columns (55 mm diameter,150 mm height and 2.32 mm thick) were packed with sieved (<2 mm) loamy sand field soil (Newport Series, FAO Class brown soil) at equivalent bulk density of 1.3 g cm^−3^. The soil was saturated with deionised (DI) water and planted with seeds of *O. sativa* spp. Azucena after germination, when the coleoptile and radical were approximately 1 cm long. Columns were kept at continuous saturation by providing DI water in a tray ponded with 2 cm of DI water. Rice plants were grown for 29 days on a 12 hour daylight cycle at 28°C daytime temperature and 20°C at night. Four hours before X-ray CT scanning, all columns were taken out of the water tray and not provided with any more water. For each column, gravimetric soil moisture content was measured before and after X-ray CT scanning to catalogue moisture loss. Four treatment columns were X-ray CT scanned daily for nine days to represent the full range of water contents expected during experimentation. Four control columns were treated in exactly the same way with the exception of X-ray CT scanning, to record any potential effects on plant growth incurred during repeated X-ray exposure. These unscanned control plants were removed from the controlled growth chamber and placed in the dark for the duration of X-ray CT scan time to account for the influence of removal from the growth room.

Plant root systems of the treatment and control columns were destructively sampled on day ten by carefully removing the intact soil cores from the polypropylene columns. The soil-root columns were placed in water and the soil was carefully removed from the root systems. Immediately after cleaning the root systems, root volume was measured with WinRHIZO® 2002c (Regent Instruments, Canada) scanning equipment and software. The root volume measurements were further verified by water displacement [31].

### Soil and Microbe Sample Preparation and Treatment

Four replicate columns (23 mm diameter, 70 mm height and 1.52 mm thick) were uniformly packed with a loamy sand (Newport Series, FAO Class brown soil), silty loam (Batcome Series, FAO Class chromic luvisol) and clay loam (Worcester Series, FAO Class argillic pelosol) to a dry weight bulk density of 1.2 g cm^−3^, saturated with sterilised DI water and gravimetrically drained to field capacity. The water status of the columns was maintained at field capacity (determined by weight) throughout the investigation by sterile deionised water addition every 1–2 days. The columns were incubated for 24 weeks at 16°C and a sub-section repeatedly scanned at weeks 0, 2, 4, 8, 16 and 24 of incubation. Scanned and unscanned soils were destructively harvested and microbial biomass carbon assessed at the end of the incubation period by chloroform fumigation extraction [32]. A value of 0.45 was selected as the conversion coefficient of ‘chloroform-labile’ carbon to microbial biomass carbon [33].

### X-ray CT Imaging

A Phoenix Nanotom X-ray CT scanner (GE Sensing and Inspection Technologies, GmbH, Wunstorf, Germany) with a tungsten transmission target obtained 3-D images of each sample column including soil and root structure where applicable. Scan settings for the plant and microbial samples are detailed in Table 1. The spot size was approximately 3 µm (Mode Zero). Datos|x 2.0 (GE Sensing and Inspection Technologies, GmbH, Wunstorf, Germany) was used to reconstruct the X-ray CT images into a 3-D volume. Individual adjustments were made for minor sample displacement during scanning.

**Table 1:** X-ray CT scan parameters.

Sample	kV	µA	Filter	Source to sample distance (cm)	Time each scan (min)	Total number of scans per sample	Voxel size (µm)
Rice in soil	110	320	0.2 mm Cu	21.5	73	9	57.3
Soil microbes	120	100	0.1 mm Cu	5.5	33	6	12.38

### Statistical Analysis

Genstat 15.1 (VSN International Ltd., UK) was used to perform an analysis of variance, containing time and all possible interactions as explanatory variables. Normality was tested by interpreting the plots of residuals; in all cases the data were normally distributed, satisfying the assumptions underlying general analysis of variance.

## Results

### Literature Analysis

In total, 126 publications were identified that related to plant and/or soil studies involving X-ray CT or X-ray exposure. Three papers (2.4% of total number analysed) using X-ray CT to visualise soil and plant samples explicitly reported the estimated dose received by the sample. The required information for estimating dose using Rad Pro is i) X-ray energy, ii) X-ray current, iii) distance from source to centre of sample, iv) thickness and type of filter used and v) total exposure time. Fewer than 5% of the publications analysed in this research area contained all the required information to calculate the X-ray dose of each sample. This is primarily due to exclusion from the reports of source to sample distance or filters used. These studies and those with minimum information (X-ray voltage, current and total scan time) to estimate dose using the Rad Pro calculator are presented in Figure 1.

The radiation dose in X-ray CT studies involving plants and microbes in soil generally are an order of magnitude lower than those considered to influence plant growth (33 Gy, Figure 1). From our extensive literature search a value for the influence of X-ray dose on soil microbial populations could not be found, although we envisage that the value that soil microbial populations could sustain would be significantly higher than the 33 Gy suggested for plants. The majority of reported studies involved doses that were lower than the 33 Gy threshold considered to significantly influence plant growth. It should be noted that for plant species considered more sensitive to the influence of X-ray radiation exposure such as field bean [23] and date palm [34], the thresholds for negative influence on growth were 26 Gy and 0.05 Gy respectively.

Prior to 1950, studies investigating the effect of X-ray exposure on plants involved direct exposure of the seed or plant to X-ray radiation (Figure 1). After 1945 there was a sharp decline in studies relating to X-ray dose on plants that included the required information to calculate dose. With the advent of X-ray CT developed by Hounsfield [35], studies involving soil microorganisms were undertaken as the possibility for non-destructive imaging of the physical structure of soil became possible. Additionally, X-ray CT was adopted for visualisation of plant root development in soil. Since 2008, technological advancements in X-ray detectors and data storage have contributed to the increase in the number of plant and soil investigations that include the use of X-ray CT.

### Impact of Repeated Scanning on Plant Roots in Soil

**Figure 2:**
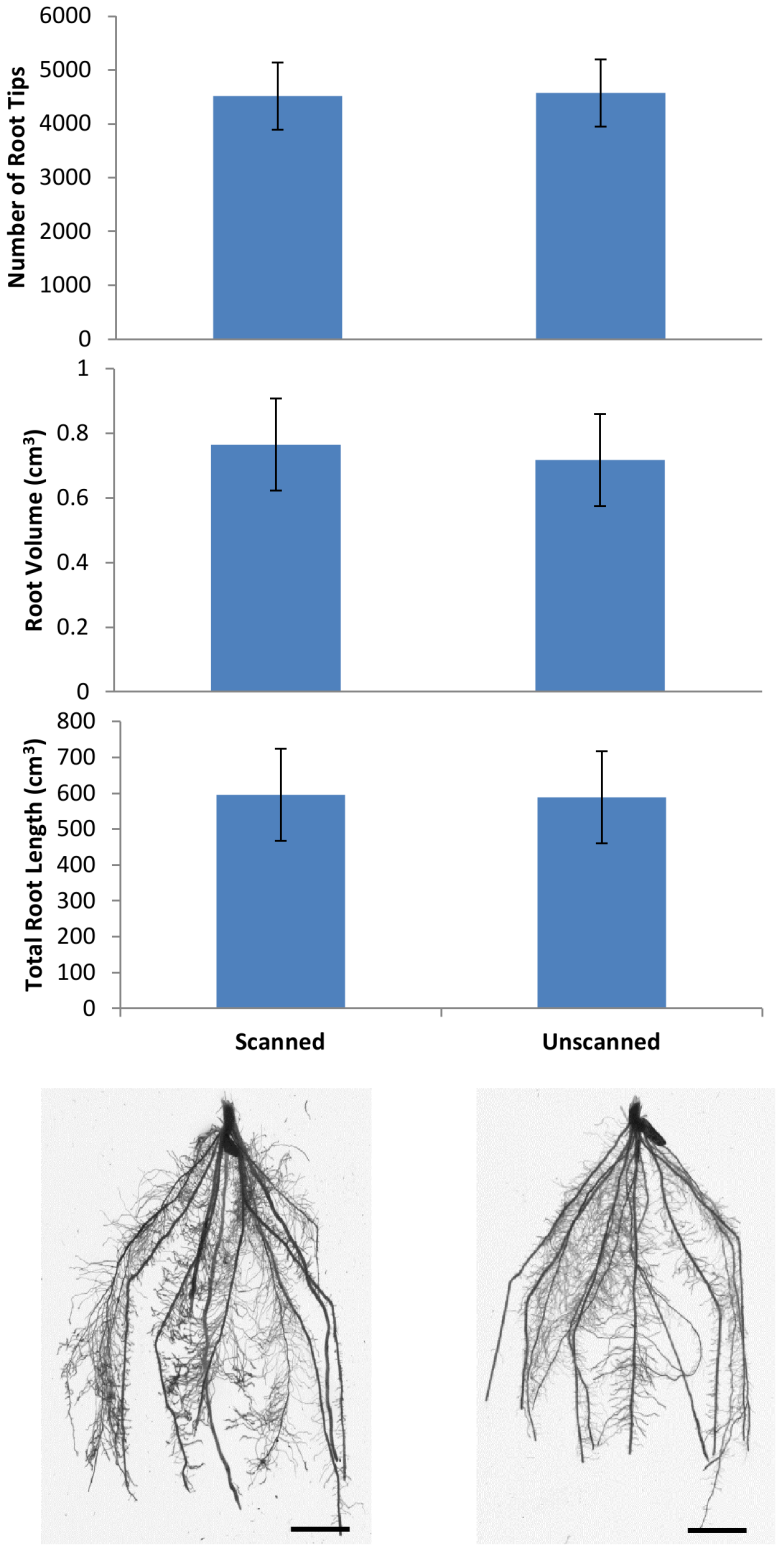
Impact of X-ray CT on rice root growth. Twenty-eight day old rice plants grown in soil were X-ray CT scanned daily for nine days. After day nine of scanning, root systems were destructively sampled via root washing and root volume was measured in WinRHIZO. Repeated exposure to X-rays had no significant effect on the number of root tips, root volume or total root length of rice grown in soil when compared to unscanned plants. Error bars depict standard error of four replicates. Total dose received by each sample was 13 Gy over nine scans. Scale bar represents 1 cm.

For the study involving rice roots growing in soil, no significant difference was found between scanned and unscanned number of root tips, root volume and total root length measured in WinRhizo (Figure 2). Scanned root systems had an average of 4512 root tips and unscanned root systems had an average of 4571 root tips (*P* = 0.928). Average root volume was 0.765 cm^3^ for scanned plants and 0.718 cm^3^ for unscanned plants (*P* = 0.752). Total root length averaged 596 cm and 589 cm for scanned and unscanned plants respectively (*P* = 0.960). The dose received by each column for a single scan was 1.4 Gy, equating to a total dose per column of 13 Gy over the ten day investigation (nine scans). Results are consistent with previous studies [1], [9], who found no significant alteration to root development in cereals at doses of 0.7 Gy and 1 Gy respectively.

### Impact of Repeated Scanning on Microbes in Soil

**Figure 3:**
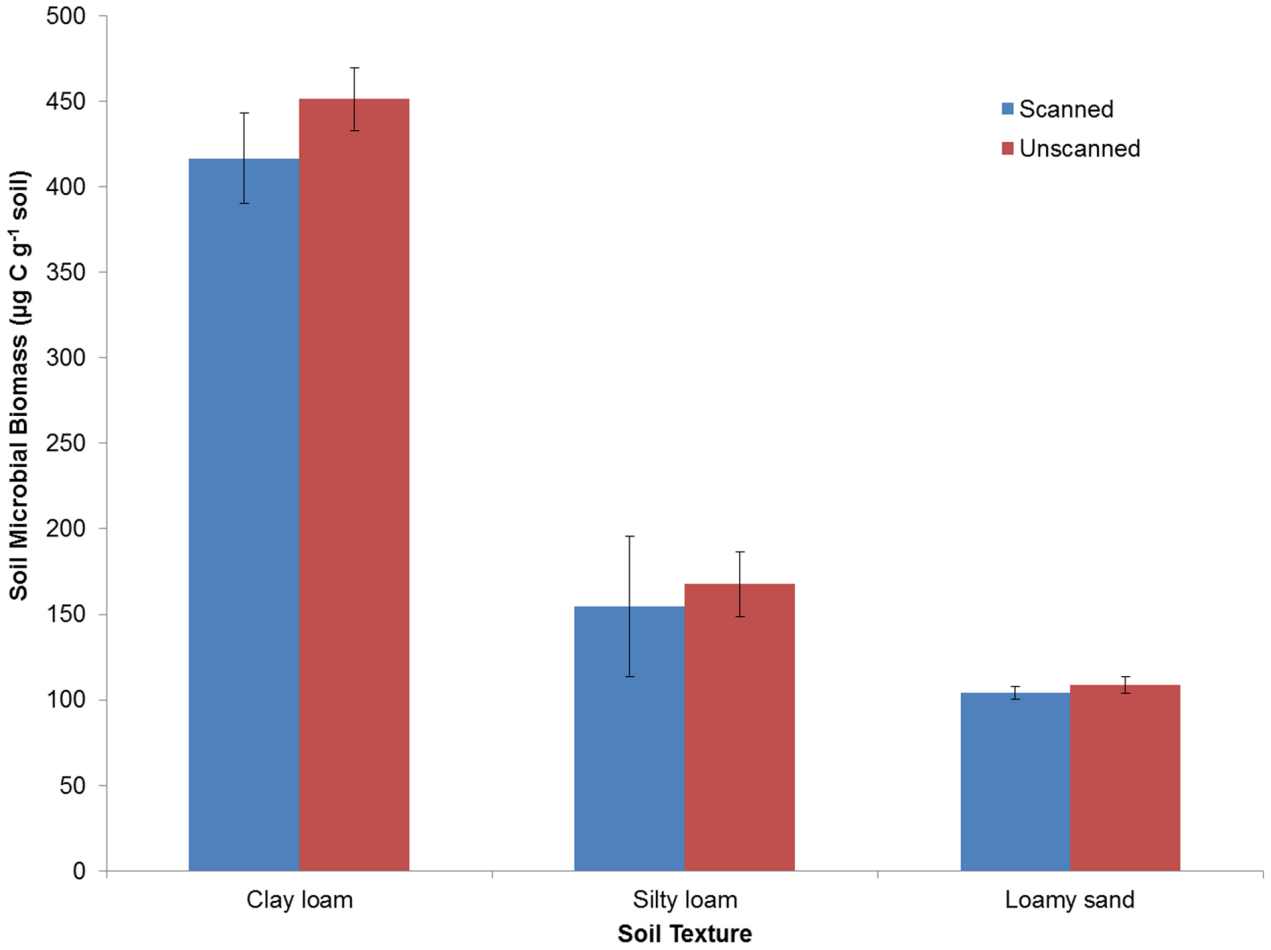
Influence of X-ray CT on soil microbial biomass after 24 weeks of incubation. Microbial biomass was measured with chloroform fumigation and compared between X-ray CT scanned columns and unscanned controls. Total dose received by each sample was 23 Gy over six scans. Error bars depict the standard error of the four replicates.

The soil microbe experiment involved scanning over a total of 24 weeks with six scanning sessions. No significant difference was found between the scanned and unscanned microbial biomass values after 24 weeks (Figure 3; *P* = 0.975). Interestingly mean biomass values were consistently higher in unscanned compared to scanned treatments across all soil textures (mean values of 451.30 and 416.66 µg C g^−1^ soil in the clay loam, 167.57 and 154.32 µg C g^−1^ soil in the silty loam and 108.71 and 104.19 µg C g^−1^ soil in the loamy sand for unscanned and scanned respectively), although none were statistically significant. Total dose received by each sample was 23 Gy over six scans.

## Discussion

Hypotheses about the hormetic effects of X-ray radiation on plant productivity drove initial investigations into the impact of X-ray dose on crops such as wheat [36] and potato [22]. There was agreement that large X-ray doses impaired plant growth and development; the disagreement arose surrounding the beneficial effects of moderate X-ray doses [37]. Eventually, researchers found that any improvement in growth rate or yield observed in very young plants dissipated at later growth stages [14]. This lack of financial benefit for crops could explain the decline in interest regarding the effects of X-rays on plants that occurred in the late 1930s (Figure 1). In the late 1980s/early 1990s, there was a resurgence of use of X-rays in plant and soil research. This likely occurred due to development of X-ray CT as a 3-D visualisation tool. X-ray CT provided another option for researchers to visualise plant root architecture in soil as well as soil structure and pore geometry.

As X-ray CT becomes more widely adopted in the plant and soil sciences, the demand for greater throughput, larger samples (to analyse field soil cores) and higher resolution will intensify. Advancements in detector technology, computer processing and data storage could both increase and decrease dose received by samples scanned using X-ray CT. For example, reduced scan times will mean that single samples are subject to less X-ray dose per scan. However, less total X-ray scan time means that researchers can complete a set of samples in the time it would have taken for one sample. We envisage that future experiments are more likely to include repeated scanning of the same sample and thus have a total dose much higher than that of a single scan. This is especially true for experiments analysing root phenotypes or soil structural development that investigate changes in these systems over time. Therefore we suggest that experimental studies utilising X-ray CT report the dose received by the sample, or at least the constituent parameters in order to allow others to make informed decisions as to the dose that experimental samples have received.

At present, those studies that have included unscanned controls have reported no significant influence of X-ray CT scanning at moderate energy levels and relatively small (<30 cm) source to sample distances. However, the vast majority of publications do not mention dose or include unscanned controls in their experiments. This is not to say that unscanned controls were not included in the experiments, but they were unreported in the published articles. Therefore, unscanned controls are important as a verification method to ensure that the X-ray parameters and resulting dose are not significantly impacting the experimental treatment. Our recent studies have shown that a typical X-ray CT experiment with repeated scanning of the same sample does not have a significant negative impact on plant root development (Figure 2) or microbial activity (Figure 3) in soil.

The main potential contributor to differences between scanned and unscanned plants may be removal from growth chamber if unscanned plants are kept under controlled conditions whilst the scanned plants are not in the growth chamber. For example, total scan time can amount to several hours that a planted sample is removed from the growth room, so in turn less hours photosynthesising. Furthermore, fluctuations in temperature within the scanning chamber itself may induce changes in the moisture content of samples. To minimise this time outside of controlled growth conditions shorter scan times are encouraged, which are becoming increasingly feasible through technological development. However, this is often at the expense of decreased image quality, which may impact on feature identification in the subject of interest. Hence there is a trade-off between scan time and image quality optimisation, which varies dependent upon experimental aims. Alternatively where practical, another largely unexplored option is to set the day/night cycle opposite to real-world conditions, so that during working hours scanning can be carried out during the night cycle for the plant. To be confident that other differences between the X-ray CT laboratory conditions and the field/glasshouse/growth room (e.g. light intensity, humidity, temperature, O_2/_CO_2_ levels) are not affecting plant growth, workers could consider leaving some unscanned controls in the usual place of growth and removing other unscanned control samples. By taking both sets of samples to the X-ray CT laboratory for the duration of the scan time, before returning again to the field/glasshouse/growth room, the potential influence of X-ray exposure on plant growth and microbial populations is reduced.

To minimise received dose, samples could possibly be moved further from the source, total scan time can be reduced, or lower energies can be used. However these options all have implications for the quality of the X-ray CT image produced. Due to the relationship between the distance of the sample and the X-ray source, there is a recognised trade-off between the achievable image resolution and received dose. Moving a sample further from the source reduces the magnification of the image received at the detector, and thus limits the achievable image resolution. Additionally, contrast within the image can deteriorate when trying to minimise dose because contrast is dependent on the energy, wavelength and type and thickness of filters used.

A more complicated aspect of assessing the impact of X-ray dose on living samples is that organisms have highly variable responses to exposure. In the case of plants, Al Khayri et al. [34] found relatively small X-ray exposures of 0.25 Gy had an influence on biochemical aspects of date palm (*Phoenix dactylifera* L.) development (i.e. DNA and pigment synthesis), as well as a negative influence on root and shoot development found by Al-Enezi et al. [25]. Alternatively, Johnson [14] found that high X-ray doses (33 Gy) had little or no observable effect on Sunflowers (*Helianthus L.*). This variability further validates the need for incorporation of unscanned controls in all X-ray CT experiments. However it is worth noting that the dose currently utilised to γ-sterilise soils (a method often used as a highly successful biocide and preferable to other sterilisation procedures such as autoclaving due to having a lessened effect on soil chemical and physical properties) is 20 000–70 000 Gy [30]. This is three orders of magnitude higher than the largest doses reported in X-ray CT investigations to date. Likewise, doses required for routine sterilisation of foodstuffs and medical appliances are ca. 25 000 Gy [18].

### Conclusions

This study supports the use of X-ray CT as a means of quantifying root and soil traits, as the results show no significant impacts on observable growth parameters due to X-ray exposure at the levels used in the study. The advantage of using X-ray CT to non-invasively characterise the 3-D geometry of soil and roots is reinforced by the insignificant impact of X-rays on soil biota and root systems in our two repeated scanning investigations. The doses received by individual samples and the total dose accumulated over the period of repeated scanning were within a range of accepted values that should not significantly influence growth (<33 Gy). Of particular importance is the fact that at the settings used, multiple scans on the same sample appear to have no effect on root phenotypic traits, confirming the appropriateness of X-ray CT for high-throughput investigations given the right scan settings. As this field of research evolves, it is anticipated that further information can be gained from a greater number of researchers reporting dose received by samples and highlighting any significant alterations to expected growth patterns.

## Supporting Information

Figure S1 **Flowchart depicting meta-analysis protocol.**
(TIF)
