# Supplementary figures and images for: Effects of X-Ray Dose On Rhizosphere Studies Using X-Ray Computed Tomography
Source: PLoS One. 2013 Jun 26;8(6):e67250. doi: 10.1371/journal.pone.0067250 (PMC3693932; doi:10.1371/journal.pone.0067250)

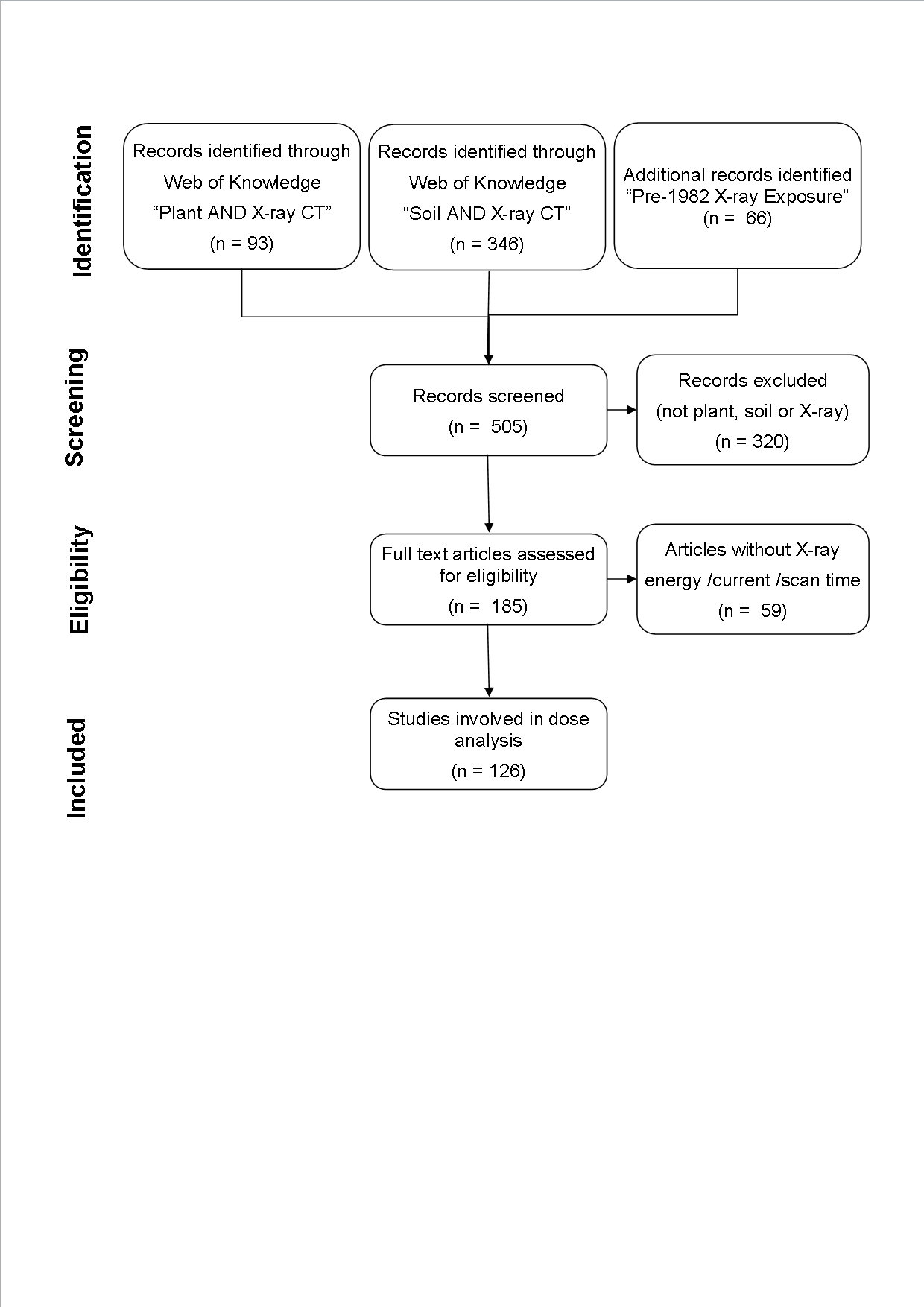

Supplement: Figure S1 — Flowchart depicting meta-analysis protocol. (TIF) [file pone.0067250.s001.tif]
